# Preparation of CNF/PDMS Superhydrophobic Coatings with Good Abrasion Resistance Using a One-Step Spray Method

**DOI:** 10.3390/ma13235380

**Published:** 2020-11-26

**Authors:** Jingda Huang, Peihao Cai, Mengmeng Li, Qiang Wu, Qian Li, Siqun Wang

**Affiliations:** 1 School of Engineering, Zhejiang A&F University, Hangzhou 311300, China; hjd1015@163.com (J.H.); caiph1998@163.com (P.C.); Limm0280@163.com (M.L.); wuqiang@zafu.edu.cn (Q.W.); liqian_polymer@126.com (Q.L.); 2 Center for Renewable Carbon, University of Tennessee, Knoxville, TN 37996, USA

**Keywords:** cellulose nanofibers, PDMS, superhydrophobic coating, abrasion resistance

## Abstract

Complex preparation methods and weak mechanical properties of superhydrophobic coatings hinder their applicability. To address these problems, cellulose nanofibers (CNFs) were used as structural materials to augment the roughness properties, while polydimethylsiloxane (PDMS) was used as the adhesive. Based on the results of previous studies, superhydrophobic coatings with good mechanical properties can be prepared by spraying the mixture onto a substrate surface; herein, the mixture comprised modified CNFs and PDMS. The resulting coating possessed excellent superhydrophobicity, which allowed a maximum water contact angle (WCA) of 158°. Furthermore, it exhibited great knife-scratch-resistance properties and good abrasion performance, which was evaluated by abrading with 800-grit sandpaper for 19 cycles (abrasion length of 380 cm) under a 100 g load. Based on the simple operation and abrasion resistance, the coating shows great potential for practical application.

## 1. Introduction

Superhydrophobic bionic technology is developing rapidly because of the excellent properties that facilitate its utilization in waterproofing, anti-fouling, drag-reducing, and self-cleaning applications [1]. Superhydrophobic coatings exhibit a water contact angle (WCA) of greater than 150° and a slide angle (SA) of less than 10° [2,3]; however, there are two basic conditions to satisfy, namely, reasonable roughness and low surface free energy [4]. Artificial superhydrophobic coatings are commonly realized by changing their surface wettability. Thus far, numerous methods have been employed to build superhydrophobic coatings, such as templating [5], etching [6], sol-gel methods [7], chemical vapor deposition [8], electrospinning [9], and spraying [10]. However, most structural materials for superhydrophobic coatings are produced with inorganic particles [11,12,13,14,15]. For example, Xue et al. used a silane coupling agent for modifying SiO_2_ nanoparticles; subsequently, the modified SiO_2_ nanoparticles were sprayed onto the substrate surfaces to fabricate superhydrophobic coatings [16]. TiO_2_ and ZnO nanoparticles have been commonly used to build superhydrophobic coatings. Furthermore, a superhydrophobic fabric was obtained using TiO_2_ particles that were grown in situ on textile fabric by a hot-pot reaction to increase the surface roughness; subsequently, these were modified to exhibit low energies using silane [17]. Siddaramanna et al. first modified ZnO particles to exhibit hydrophobic properties and then directly attached these particles to an aluminum alloy surface to prepare a superhydrophobic coating [18]. However, these substances are non-renewable materials.

Cellulose nanofibers (CNFs) are mainly derived from biomass materials and are prepared by employing mechanical polishing and acid hydrolysis using plant fibers and organic polymers. Furthermore, they exhibit a large length-to-diameter ratio, a large specific surface area, and excellent biocompatibility [19,20,21]. Numerous studies have elucidated the application of CNF to superhydrophobic materials. For example, CNF aerogels with high elasticity could be prepared by freeze-drying an aqueous suspension of CNF, which is then modified with octyl trichlorosilane using chemical vapor deposition (CVD) to achieve water–oil separation [22]. Similarly, superhydrophobic CNF/PVA composite aerogels were made from an aqueous suspension of CNF/PVA and treated with methyl trichlorosilane by CVD; PVA improved the strength and toughness of the aerogel [23]. CNF has a high flexibility and a high length-to-diameter ratio, thereby allowing it to easily form a network structure, which is advantageous for augmenting the surface roughness of superhydrophobic coatings. Furthermore, the presence of several active hydroxyl groups on the surface render it beneficial for low surface energy modification [24]. In a previous study, our research group made some progress on the preparation of superhydrophobic coatings using CNFs; after low surface energy modification, the CNF or cellulose nanocrystal (CNC) was incorporated onto the substrate surface, which was treated with adhesives in advance to obtain the superhydrophobic coating [25].

The poor abrasion resistance of superhydrophobic coatings has impeded their development [26,27]. A superhydrophobic coating could be prepared by immersing a small piece of wood in a suspension of the modified SiO_2_ nanoparticles and subsequently dried; however, the poor binding force between the particles and the wood surface leads to poor abrasion resistance [28]. Tu et al. tried using epoxy resin to improve the adhesion between SiO_2_ nanoparticles and the substrate, but the effect was observed to be inconspicuous [29].

**Figure 1:**
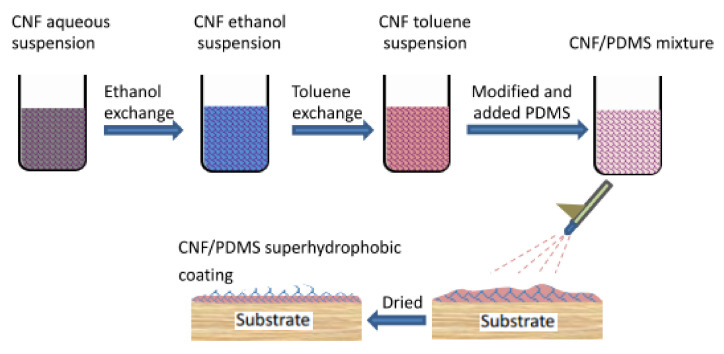
Preparation process of the cellulose nanofiber/polydimethylsiloxane (CNF/PDMS) superhydrophobic coating.

Therefore, polydimethylsiloxane (PDMS) was used as an adhesive in this study to increase the adhesion between the superhydrophobic coating and the substrate. After curing, PDMS not only has good adhesive force, but it also acts as a non-rigid colloid; this beneficially increases the abrasion resistance according to our previous research [30]. Moreover, as PDMS itself is a kind of hydrophobic material, it has the additional advantage of achieving a degree of uniformity of the low surface energy on the coating surface. The CNF or CNC superhydrophobic coatings in our previous research were prepared using a convoluted process [25]. In this study, the superhydrophobic surface was prepared by a one-step spray method. Figure 1 shows the preparation process of the CNF/PDMS superhydrophobic coating. CNF was modified and mixed with PDMS to form a hydrophobic mixture, and then a CNF/PDMS superhydrophobic coating was prepared by one-step spray and dried. This study will be beneficial to those investigating the theory that governs the industrialization of CNFs. Furthermore, the results obtained will expand the applicability of CNFs and elucidate their value. 

## 2. Results and Discussion

### 2.1. Formation Mechanism

**Figure 2:**
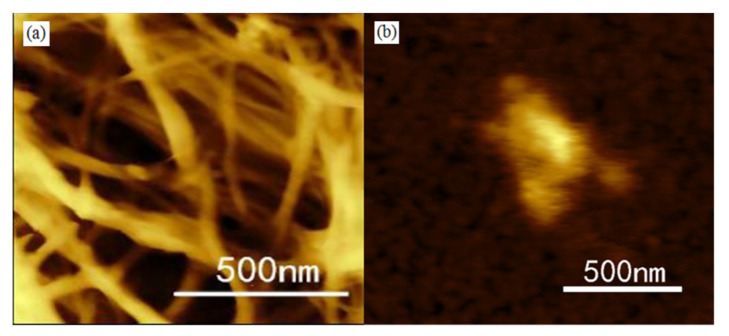
AFM images of CNF (**a**) before modification and (**b**) after modification.

As shown in Figure 2a, CNF before modification was filamentous. However, CNF after modification was aggregated due to mutual entanglement of CNF during modification, which is proven in the following Surface Morphologies section.

A feasible way to prepare the CNF/PDMS superhydrophobic coatings was achieved by spraying the CNF/PDMS mixture onto the substrate surfaces, with the hydrophobic PDMS serving as the adhesive. In contrast, if a hydrophilic adhesive was used, a minimum of two steps was needed to prepare the superhydrophobic coating. As described in our previous study [25], the hydrophilic adhesive (a commercial quick-drying transparent topcoat) was applied to the wood surface, and the hydrophobic-modified CNC was then sprayed onto the adhesive surface. 

**Figure 3:**
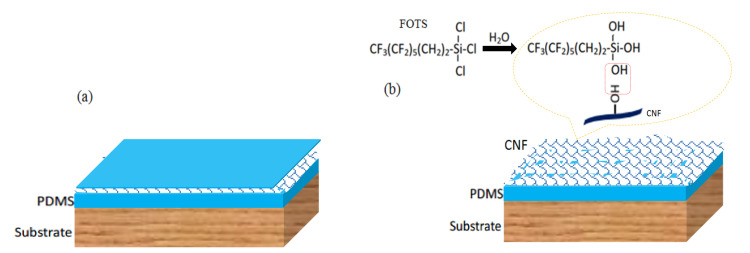
(**a**) Schematic of CNF/PDMS coating with excessive PDMS and (**b**) formation mechanism of the CNF/PDMS superhydrophobic coating.

In the CNF/PDMS mixture, PDMS itself is hydrophobic, and its concentration is a key factor. If the dosage is too high, after the drying process, the resulting PDMS layer could be too thick and cover most or all of the CNF, leading to loss of superhydrophobicity (as shown in Figure 3a). Moreover, an excessively low dosage would cause the resulting PDMS layer to become too thin and provide insufficient adhesion, leading to easy removal of the CNF/PDMS coating under the action of an external force. The ideal result is that the PDMS is able to support a certain degree of adhesion, while not absolutely covering the CNF modified using FOTS (as shown in Figure 3b). Through optimization experiments, the mass ratio of the PDMS and the CNF toluene suspension was determined to be 1:19 in this study; i.e., 5 wt% of PDMS is appropriate for the preparation of the CNF/PDMS superhydrophobic coating. Therefore, the following points have been established: (1) the superhydrophobic coating can be successfully prepared using a one-step spray method; (2) the process does not require the use of inorganic particles; (3) the wear resistance of the superhydrophobic coating is improved; (4) CNF is an environmentally benign biomass material.

### 2.2. Wettability

**Figure 4:**
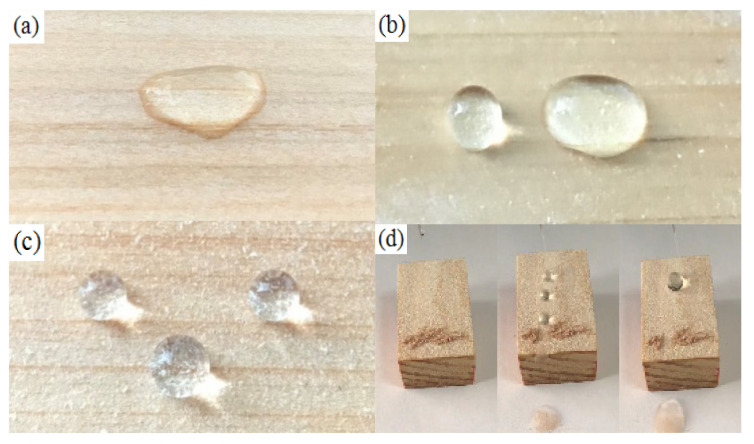
(**a**) Images of the untreated wood, (**b**) pure PDMS coating, (**c**) CNF/PDMS coating, and (**d**) self-cleaning process of the CNF/PDMS coating.

Figure 4a shows that the untreated substrate surface is hydrophilic because it comprises a large number of hydrophilic hydroxyl groups. Accordingly, the wood was easily affected by the humidity of the surrounding environment, while undergoing continuous expansion and contraction. After being treated with PDMS, as shown in Figure 4b, the WCA of the sample surface was only 123°, therefore not superhydrophobic. When treated using CNF/PDMS (as shown in Figure 4c), the WCA of the sample was observed to be as high as 158°, while the maximum SA was 7°; this confirms the superhydrophobicity of the coating. This result was observed because the wood surface with a high surface free energy was covered by the CNF/PDMS coating with low surface free energy. Furthermore, the surface has a reasonably rough structure that would be formed by the mutual intertwining and stacking of CNFs, thereby meeting the two basic conditions of superhydrophobicity. Self-cleaning is a typical characteristic of a superhydrophobic surface. As shown in Figure 4d, when the sample was tilted by 8° with respect to the horizontal line, the droplets were observed to push the dust away.

### 2.3. Surface Morphologies 

**Figure 5:**
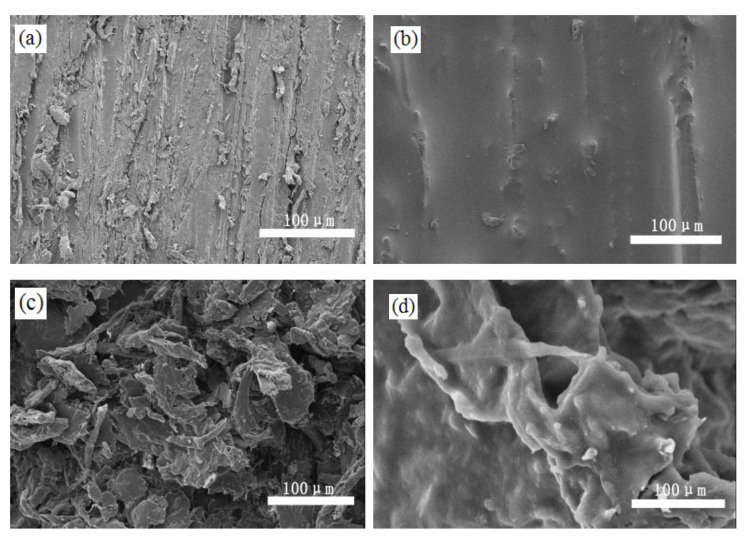
SEM images of (**a**) wood surface, (**b**) pure PDMS coating, and (**c**) CNF/PDMS coating and (**d**) its high-magnification image.

CNF is a filamentous material with a high length-to-diameter ratio. These dimensions are the reason why the CNF can form a rough convex structure by mutual entanglement [10]. Figure 5a shows the surface structure of the untreated wood, where numerous grooves were formed due to the destruction of wood vessels during cutting or sanding; these wood vessels could provide a certain degree of rough structure, but this was not enough to make the surface superhydrophobic, even if the low surface free energy modification was conducted according to our previous research [10]. When the wood surface was only covered by PDMS (as shown in Figure 5b), these grooves were filled and the surface became smooth as compared to that of the untreated wood; however, superhydrophobicity was not achieved (as shown in Figure 4b). As shown in Figure 5c, after being treated with the CNF/PDMS mixture, the coating surface showed that parts of the CNF on the PDMS formed into a microscale aggregation by mutual entanglement, which was a result of the hydrophobic modification and spraying. Figure 5d is a high-magnification image of the CNF aggregation, wherein the nanoscale denticulation of the aggregated form was observed. The microscale CNF aggregation and its nanoscale denticulations were the reasons for realizing superhydrophobicity.

### 2.4. Chemical Composition Analysis 

**Figure 6:**
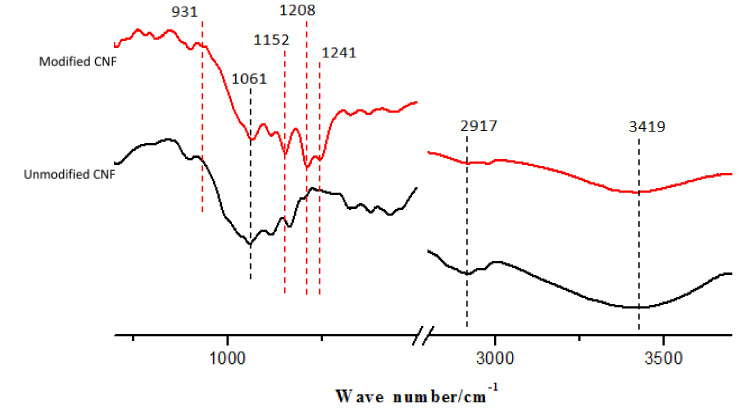
FTIR spectrum of the CNF before and after hydrophobic modification.

As shown in Figure 6, the FTIR absorption peaks (black dotted line) observed for the CNF before and after hydrophobic modification were attributed to the vibrations of the C-OH bond, C–H rings, and the side group (1061 cm^−1^ peak); the peak at 2917 cm^−1^ formed due to the symmetrical stretching of C–H. The peak at 3419 cm^−1^ was observed due to the hydroxyl groups on the CNF surface; however, most of the hydroxyl groups were removed after modification, resulting in a weaker strength than that observed before modification. The new peaks (red dotted line) at 931 cm^−1^ observed for the CNF after modification resulted from the stretching vibrations of Si-O bonds, which were sourced from FOTS and the hydroxyl groups of the CNF surface. The stretching vibration of C–F on FOTS, which provided a low surface free energy, exhibited peaks at 1152 , 1208, and 1241 cm^−1^.

### 2.5. Sandpaper Abrasion Test 

At present, sandpaper abrasion is the most commonly used test method for evaluating the mechanical properties of superhydrophobic coatings; this method has been conducted as described in our previous study [4]. Briefly, the sample was placed with the coating face down on sandpaper and moved 10 cm; it was then moved another 10 cm after the sample was rotated by 90°. This completed one abrasion cycle; the test was continued until the WCA was below 150°. 

**Figure 7:**
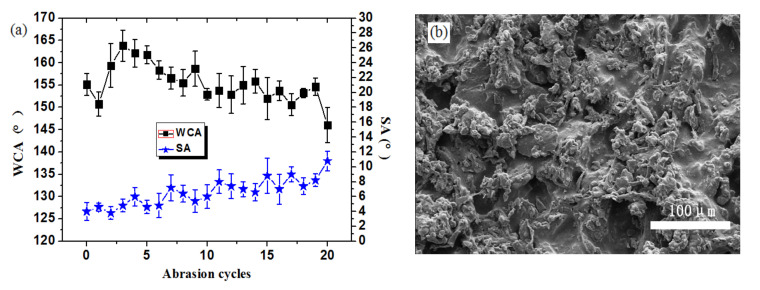
(**a**) Graphical representation of the water contact angle (WCA) and slide angle (SA) of the CNF/PDMS coating during sandpaper abrasion and (**b**) SEM image of CNF/PDMS coating after sandpaper abrasion.

According to our previous research, the pure CNF superhydrophobic coating appeared to be significantly damaged after only one abrasion cycle, which resulted in the exposure of the hydrophilic wood surface and the loss of superhydrophobicity [10]. When the load was 100 g and an 800-grit sandpaper was used (Figure 7a), the CNF/PDMS superhydrophobic coating was not destroyed, and its WCA did not fall below 150° until after 19 sandpaper abrasion cycles. This proved that PDMS contributed to good adhesion properties between the CNF and wood. During the abrasion process, the WCAs gradually reduced and the SAs continually increased. This was because the CNF aggregation was destroyed or removed during sandpaper abrasion. Distinct from the pure CNF coating, even when the CNF layer in the CNF/PDMS sample was removed, the PDMS layer remained, and the maximum WCA of the surface was still 129° because of the hydrophobicity of PDMS. As shown in Figure 7b, after 20 abrasion cycles the CNF particles were destroyed, and the contact area between the water droplet and sample surface became larger, leading to a low WCA and high adhesion of water droplets (i.e., high SA). 

**Figure 8:**
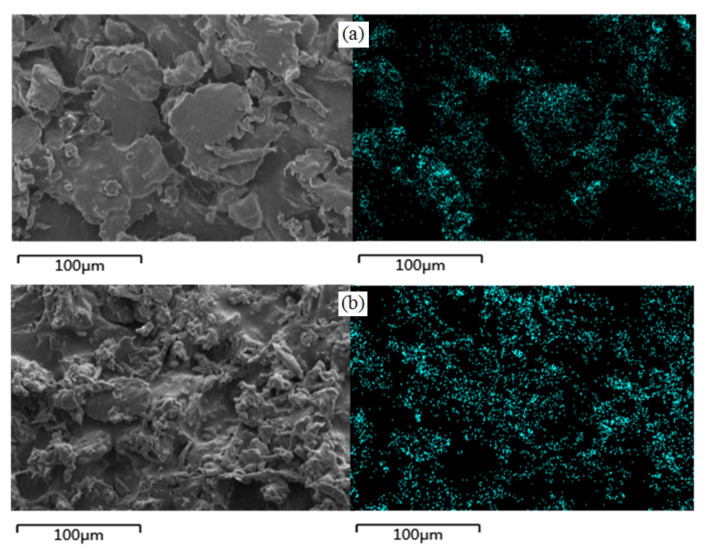
F elemental mapping of the CNF/PDMS coating (**a**) before and (**b**) after sandpaper abrasion.

As is known, superhydrophobicity depends on a reasonably rough structure and low surface free energy. Here, CNF was modified with FOTS, and the low surface free energy was mainly governed by the F element of FOTS. To further confirm the reason for the loss of superhydrophobicity, an energy-dispersive spectrometer (EDS) was used to analyze the change in the chemical components; the EDS images of the F element for the CNF/PDMS samples are shown in Figure 7, before and after 20 abrasion cycles. The F element was uniformly filled up in all CNF areas before sandpaper abrasion (as shown in Figure 8a), proving that FOTS could be adequately grafted to the CNF surface. After sandpaper abrasion was performed for 20 cycles, the F element in the remaining CNF areas was still equally distributed (as shown in Figure 8b), proving that the F element on the CNF surface was not affected by sandpaper, and the loss in superhydrophobicity was a result of destruction in the rough structure.

### 2.6. Knife-Scratch Tests

**Figure 9:**
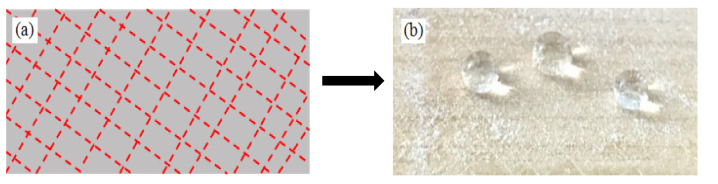
(**a**) Schematic of CNF/PDMS coating and (**b**) wettability of CNF/PDMS coating after knife scratches.

Knife scratches have been observed to occur frequently during the use of superhydrophobic coatings. To systematically study the influence of the scratch size on superhydrophobic coatings, knife scratches were applied to the samples (as shown in Figure 9a) using a knife with a 0.5 mm thickness. The results are shown in Figure 9b: water droplets easily rolled across the scratches, and the superhydrophobicity was not affected. This is because water droplets have an average volume of ~50 μL and are therefore larger than the width of the scratches, which are small as a result of the elasticity of the PDMS.

## 3. Materials and Methods 

### 3.1. Materials

Polydimethylsiloxane (PDMS, Sylgard 184 silicone elastomer) and its curing agent were purchased from Dow Corning, Inc, Midland, MI, USA. Cellulose nanofiber (CNF) water suspension without modification (white paste, 1.03% solid content, purity >99%, and a length of approximately 1–5 μm and a diameter of approximately 5–100 nm) was purchased from Tianjin Woodelf Biotechnology Co. Ltd, Tianjin, China. Softwood bleached sulphate fiber pulp, which was made from spruce wood, was used as source material to prepare CNF by mechanical milling using a supermasscolloider (Masuko sangyo Co., LTD., Kawaguchi/Saitama, Japan). 1H,1H,2H,2H-Perfluorooctyltrichlorosilane (FOTS, CF3(CF2)5(CH2)2SiCl3, 97%) was purchased from Sigma Aldrich, China. Both anhydrous ethanol (>99.5%) and toluene (>99.8%, AR) were purchased from Alfa Aesar, China. Small pieces of wood (dimensions 4.5 cm × 2.5 cm × 1.5 cm) were used as substrates.

### 3.2. Preparation of CNF/PDMS Superhydrophobic Coating

#### 3.2.1. Organic Solvent Exchange

As described in our previous study [4], to achieve maximum removal of water the purchased CNF aqueous suspension was centrifuged with a high-speed centrifuge at 12,000 rad/min. Subsequently, the collected CNF was dispersed by stirring into the same volume of anhydrous ethanol as that of the collected water. This process was deemed to be one cycle; herein, four cycles were performed. The modification of CNF necessitates the use of toluene as the medium; therefore, after the anhydrous ethanol exchange, CNF was dispersed into toluene. Subsequently, the same operation as that outlined for ethanol exchange was conducted to achieve the toluene exchange and form the CNF suspension in toluene; the concentration of this suspension was adjusted to 1.0 wt%. 

#### 3.2.2. Hydrophobic Toluene Suspended CNF

The 1.0 wt% CNF toluene suspension was poured into a glass container, to which FOTS was added; the volume ratio of the FOTS and CNF toluene suspension was 1:50. Subsequently, the glass container was sealed, and the mixture was stirred ~4 h using a magnetic stirrer. 

#### 3.2.3. Spraying 

The PDMS and the curing agent (mass ratio of 10:1) were added to the modified CNF toluene suspension; the mass ratio of PDMS and CNF toluene suspension was 1:19. Subsequently, stirring was performed for 30 min to form a uniform mixture. The mixture was poured into an airbrush with a nozzle diameter of 0.5 mm (Uxcell mini 0.5 K3 HVLP gravity feed airbrush paint spray gun, Aotl Tools Guangzhou Co.,LTD., China) and sprayed onto the substrate surfaces at a pressure range of 10–30 Mpa; the pressure came from a pint-sized air compressor. A spray quantity of ~0.5 g was used on the spray area (4.5 cm × 2.5 cm), and the distance from the substrates to the airbrush nozzle was about 30 cm. Finally, it was dried for 2 h at 100 °C to synthesize completely pure PDMS.

### 3.3. Characterization 

Scanning electron microscopy (SEM) images of different sample surfaces were observed using a Zeiss Auriga SEM/FIB crossbeam workstation (Jena, Germany). The chemical changes in the CNF were analyzed before and after modification via an attenuated total reflection Fourier transform infrared spectrometer (ATR-FTIR, Perkin Elmer, Waltham, MA, USA, range from 600 to 4000 cm^−1^ with 16 scans), and the degree of crystallinity was elucidated by using X-ray diffraction (XRD). Thermogravimetric analysis (TG) was used to analyze the CNF before and after modification. 

As described in our previous study, the WCA of the sample surfaces was measured using a commercial contact angle meter (Shanghai Zhongchen JC2000D, China) with a rotatable sample platform that was able to measure the SA. The liquid droplets used for measurement exhibited a volume of 4–8 μL, and the average values of five measurements at different locations were obtained for each WCA or SA.

## 4. Conclusions

CNF/PDMS superhydrophobic coatings could be prepared using a one-step spray method. The resulting coating showed excellent superhydrophobicity and mechanical properties. Hydrophobic-modified CNF and PDMS were optimally mixed to solve the problem of spraying the CNF and PDMS separately. PDMS with good elasticity played an important role in superhydrophobicity and the wear-resistance mechanism of the CNF/PDMS coatings. Based on a simple preparation method and good mechanical properties, the CNF/PDMS superhydrophobic coatings exhibit significant potential for practical applications.
